# Unique *k*-mers as Strain-Specific Barcodes for Phylogenetic Analysis and Natural Microbiome Profiling

**DOI:** 10.3390/ijms21030944

**Published:** 2020-01-31

**Authors:** Valery V. Panyukov, Sergey S. Kiselev, Olga N. Ozoline

**Affiliations:** 1 Institute of Mathematical Problems of Biology RAS—the Branch of Keldysh Institute of Applied Mathematics of Russian Academy of Sciences, 142290 Pushchino, Russia; panyukov@itaec.ru; 2 Structural and Functional Genomics Group, Federal Research Center “Pushchino Scientific Center for Biological Research of the Russian Academy of Sciences”, 142290 Pushchino, Russia; anthyllium@gmail.com; 3 Institute of Cell Biophysics of the Russian Academy of Sciences, 142290 Pushchino, Russia

**Keywords:** bacterial genomes, genome barcodes, *k*-mers, alignment-free algorithms, phylogenetic trees, metagenomes, taxonomic profiling, phylotyping, human microbiome

## Abstract

The need for a comparative analysis of natural metagenomes stimulated the development of new methods for their taxonomic profiling. Alignment-free approaches based on the search for marker *k*-mers turned out to be capable of identifying not only species, but also strains of microorganisms with known genomes. Here, we evaluated the ability of genus-specific *k*-mers to distinguish eight phylogroups of *Escherichia coli* (A, B1, C, E, D, F, G, B2) and assessed the presence of their unique 22-mers in clinical samples from microbiomes of four healthy people and four patients with Crohn’s disease. We found that a phylogenetic tree inferred from the pairwise distance matrix for unique 18-mers and 22-mers of 124 genomes was fully consistent with the topology of the tree, obtained with concatenated aligned sequences of orthologous genes. Therefore, we propose strain-specific “barcodes” for rapid phylotyping. Using unique 22-mers for taxonomic analysis, we detected microbes of all groups in human microbiomes; however, their presence in the five samples was significantly different. Pointing to the intraspecies heterogeneity of *E. coli* in the natural microflora, this also indicates the feasibility of further studies of the role of this heterogeneity in maintaining population homeostasis.

## 1. Introduction

The rapid growth of available genomic data opens up new horizons for their heuristic analysis and expands the possibilities of the approaches traditionally used in comparative genomics. Until recently, classical methods based on multiple alignment of orthologous nucleotide or amino acid sequences were the main tools in taxonomic studies, and the variable regions of evolutionarily conserved 16S rRNA genes still serve as reliable markers for genus or species identification [1,2,3]. However, the multiplicity of rRNA genes in bacterial genomes, their intraspecies homology, and intragenomic variations limit the use of rRNA-derived markers for strain identification [4,5]. Therefore, sets of the single-copy genes encoding “housekeeping proteins” were adapted for differential analysis on the intraspecies level [6]. This approach requires the availability of assembled and annotated genomes, as well as significant time and computer resources. More recently, new methods have been introduced for taxonomic analysis. They are based on the use of short oligonucleotides (*k*-mers, *n*-grams, *l*-tuples) of length *k* (*n* or *l*) and are free from the alignment stage [7,8].

Initially exploited as tools for linguistic comparison of nucleotide sequences over 30 years ago [9,10,11], *k*-mer-based approaches are now widely used in many specialized bioinformatics programs and algorithms. For example, NCBI BLAST uses *k*-mers as “seeds” in the first step of finding target sequences in databases [12,13,14]. Short oligonucleotides initiate alignment of sequence reads [15,16] and afterwards are implemented for their assembling de novo with de Bruijn graphs [17,18,19,20,21]. They were adapted for evaluating sequencing data, including quality control [22,23], SNP identification [24,25], and even for virtual sequence error correction [25,26,27,28]. Furthermore, a combination of *k*-mer analysis with machine learning algorithms allows predicting some phenotypes, for instance, antibiotic resistance [29,30,31,32]. The intensive use of *k*-mers as taxonomic markers started very recently (for a review, see [33]), but they have already been applied in several computer algorithms. 

Thus, in 2013, a strategy was developed to search for strain/species—specific 50-mers to identify microbes with diagnostic microarrays [34]. Subsequently, it was used in the Genome Specific Marker algorithm (GSMer) [35], which revealed bacterial species associated with the disease in three clinical datasets. Later, the KRAKEN algorithm was proposed, exploiting the exact alignment of *k*-mers with metagenomic DNA sequences [36]. It operates with a database that stores marker *k*-mers (31-mers by default), ascribed to the lowest common ancestor of microorganisms that contain them in their genomes. By transforming the entire genomic database into a simple look-up table, KRAKEN significantly reduced the time for comparative analysis, but required reliable phylogenetic support. Later, the program was improved due to the expanded taxonomy and the possibility of hierarchical scanning of several databases [37]. 

A “succinct” representation of genomes, in the form of *k*-mers or their hash-indexes exploit several other algorithms proposed for taxonomic analysis, including CLARK [38], MASH [39], GOTTCHA [40] HSC [41], and MetaOthello [42]. CLARK (CLAssifier based on Reduced *k*-mers) [38] applies indexed hash-tables for 31-mers to sort target samples with genomic information converted to the same format. MASH clustered all genomes from NCBI RefSeq using a locality-sensitive hashing technique [39]. GOTTCHA (Genomic Origins Through Taxonomic CHAllenge) generates FASTA files representing the “unique genomes” of reference organisms at their specific taxonomic level and operates with 30-mers for comparative analysis [40]. The high-performance short sequence classifier (HSC) [41] stores unique 15-mers in hash tables together with identifiers (IDs) of NGS sequence reads containing them. This provides an attractive possibility to combine reads into longer fragments based on the similarity of *k*-mers. 

Liu et al. [42] addressed the question of how accuracy and sensitivity depend on the *k*-mer length. The authors compared five algorithms using simulated and empirical datasets at different taxonomic levels and obtained only a small dependence on the value of *k* for both parameters. Thus, for example, the sensitivity of KRAKEN, CLARK, and MetaOthello in comparative tests performed for Illumina metagenomic data and carried out with 20-mers was 92.1 ± 0.1%, 92.55 ± 0.05%, and 92.35 ± 0.15%, respectively [42]. The percentage of reads with a correctly defined genus ranged from 94.4% to 97.2%, which is very close to the sensitivity of the 16S rRNA typing [5], while the accuracy of species identification (~85%) [42] was higher than with the classical phylotyping (65–83% [5]). This accuracy of the *k*-mer-based taxonomy made it possible to distinguish *Bacillus cereus* and *Bacillus anthracis* strains sharing 99% identity [43]. This means that *k*-mer-based approaches operating with a huge number of marker sequences can be useful not only for taxonomic, but also for phylogenetic analysis. This would be of particular importance for intraspecies taxonomy, where pathogenic strains are often very difficult to distinguish from non-pathogenic. Therefore, in this study, we updated the previously developed UniSeq software [44] for a more accurate search for unique *k*-mers in bacterial genomes and for the first time applied them to distinguish between eight phylogroups of *Escherichia coli*, classified by Clermont et al. [45,46]. 

Initially, this classification included only phylogroups A, B1, B2, and D [47]. In 2004, seven strains with serotypes O157:H7 and O55:H7 were transferred from group D into a separate group E [48], and this was accepted in later works [49,50]. In 2008, the classification was modernized and some strains of group D formed phylogroup F [51]. Around 2010-2011, it became clear that group B1 also needed to be reconsidered, and fourteen strains were allocated into group C [52], which was in line with a previous suggestion [48] based on phylogenetic features of four strains. In 2016, two new isolates of intestinal *E. coli* of *Marmota himalayana* were characterized [53]. Together with other bacteria with partially assembled genomes, they formed a branch on the phylogenetic tree between groups F and B2 and were assigned to phylogroup G [46]. Therefore, the modern version of the classification includes eight phylogroups. Here, we evaluated the ability of genus-specific *k*-mers to distinguish them using 124 genomes for phylotyping, and for the first time, used marker *k*-mers to assess the heterogeneity of *E. coli* populations in eight human microbiomes. 

## 2. Results

### 2.1. Selection of k-Values for Phylogenetic and Taxonomic Analysis

The seven largest genomes, one from each phylogroup A, B1, B2, C, D, E, and F were selected to compare the size and composition of sets containing “unique” *k*-mers, which are absent in bacteria of any genus, except *Escherichia* and *Shigella*. Only complete genomes previously assigned to particular phylogroups were selected. Since the genomes of all strains previously assigned to the phylogroup G were deposited in NCBI in contigs, a representative of this group was not included in the list at this stage. However, the most characterized genome of the laboratory strain *E. coli* K-12 MG1655 was added as the second representative of the group A. The search for unique *k*-mers with even *k*-values in the range (16 ≤ *k* ≤ 22) was carried out using the upgraded UniSeq algorithm described in the Materials and Methods section. The reference set contained the sequences of all fully assembled bacterial genomes and plasmids of the NCBI GenBank (as of March 19, 2019), from which the chromosomes and plasmids of bacteria belonging to the genus *Escherichia* and closely related *Shigella* were removed. 

**Figure 1:**
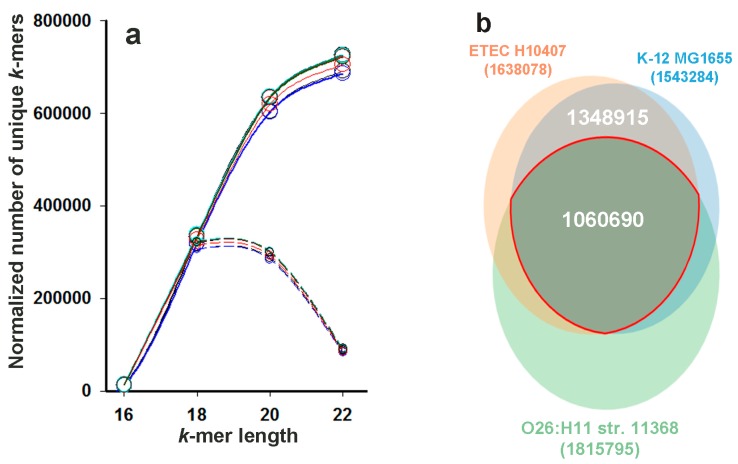
The size of the “unique genomes” represented by *k*-mers of different length for eight individual *E. coli* chromosomes, and the degree of their intersection exemplified by three indicated genomes. (**a**) The solid lines show the normalized per 1 Mbp in each genome number of *k*-mers (N), found in the chromosomes of *E. coli* (strains: K-12 MG1655, ETEC H10407, O26:H11 str. 11368, ABU 83972, APEC O78, str. 042, O157:H7 str. EC4115 and O7:K1 str. CE10) that are absent in the nucleotide sequences of the reference database. Dashed lines show the increment curves plotted for ΔN/Δ*k*. (**b**) Venn diagram illustrating the intersection between the sets of 18-mers identified in the genomes of two bacteria from group A (*E. coli* K-12 MG1655 and ETEC H10407) and the *E. coli* O26:H11 str. 11368, belonging to group B1. The number of unique 18-mers in each genome, the size of their common set and the intersection between the two sets of group A are indicated without normalization. The diagram was created using a Venn Diagram Maker [54].

As before, for the genomes of four *Enterobacter* strains, *Clostridium sporogenes* and *Cellulomonas flavigena* [44], we obtained a huge number of genus-specific *k*-mers with different lengths (Figure 1a, solid lines). Since the scanning was done with 1 bp resolution, most of the revealed sequences overlap, but all of them are equivalent and each can be considered as a taxonomic marker. This is of particular importance for the analysis of metagenomes from natural populations, where fragments belonging to hundreds and thousands of different genomes are mixed, and only sequences of dominant taxa are well represented. Therefore, it is reasonable to use long *k*-mers for taxonomic analysis, as was done earlier [34,35,36,37,38,39,40,42,43], because a large number of them increases the probability of detecting even poorly presented genomes. This number is rapidly increasing in the range from 16 to 20 bp (Figure 1a). However, for *k* ≥ 22 (Figure 1a and [44]), it reaches a plateau, and a further increase makes a relatively small contribution to the set of marker sequences. Therefore, we used the longest *k*-mer provided by the current version of UniSeq *k*-mers (22-mers) for taxonomic analysis.

Phylogenetic analysis does not require large sets of marker sequences, but may be dependent on the degree of their diversity. Since each unique *k*-mer together with flanking nucleotides in the genome gives two unique *k*+2-mers, it would be reasonable to use such marker sequences, for which *k*–2-mers are not unique, i.e., those that are “primary unique” sequences. The dashed plots in Figure 1a show that the “primary” 18- and 20-mers give almost the same contribution to cumulative plots (solid lines). However, the proportion of new 18-mers in the total set of sequences with this length is twice larger than the percentage of “primary unique” 20-mers in the corresponding set (Figure 1a). This makes sets of 18-mers more diverse, which may be more suitable for phylogenetic analysis. The Venn diagrams shown in Figure 1b illustrate the typical level of similarity between sets of species-specific *k*-mers obtained for two genomes of phylogroup A (strains K-12 MG1655 and ETEC H10407) and illustrate their higher difference from the set of marker sequences obtained for the genome of other group (strain O26:H11 str. 11368 from phylogroup B1). Such a difference between all genomes, even those that belong to the same group, allowed us to assess the ability of unique *k*-mers, selected without any bias to specific genomic loci, to distinguish phylogroups of *E. coli*. 

### 2.2. Alignment-Based Multilocus Sequence Typing Resulted in Tree with Expected Topology and Predicted New Members for E. coli Phylogroups 

The sequences of the 27 discriminatory genes listed in Supplementary Table S1 were used to obtain a phylogenetic tree based on classical multilocus typing. Thirteen of them encode enzymes of metabolic pathways (*aes*, *icd*, *pabB*, *trpA*, *trpB*, *fumC*, *mdh*, *purA*, *aspC*, *fadD*, *uidA*, *aroE*, *mtlD*); ten belong to replication, repair, or transcription systems (*polB*, *gyrA*, *recA*, *dinB*, *dnaG*, *mutS*, *arcA*, *cyaA*, *grpE*, *rpoS*), and the remaining four control the production of two symporters (*putP*, *lysP*), kinase (*adk*) and protease (*clpX*). The sequences of these genes were obtained from 124 *E. coli* genomes (listed in Supplementary Table S1), including 59 complete genomes, whose phylogroups were previously identified in original papers [45,52,55,56,57,58,59,60,61,62,63,64,65,66,67,68,69] (shown in bold in Supplementary Table S1). The remaining 65 genomes unassigned to phylogroups and containing orthologs of discriminatory genes were added to increase the resolution of phylogenetic trees.

**Figure 2:**
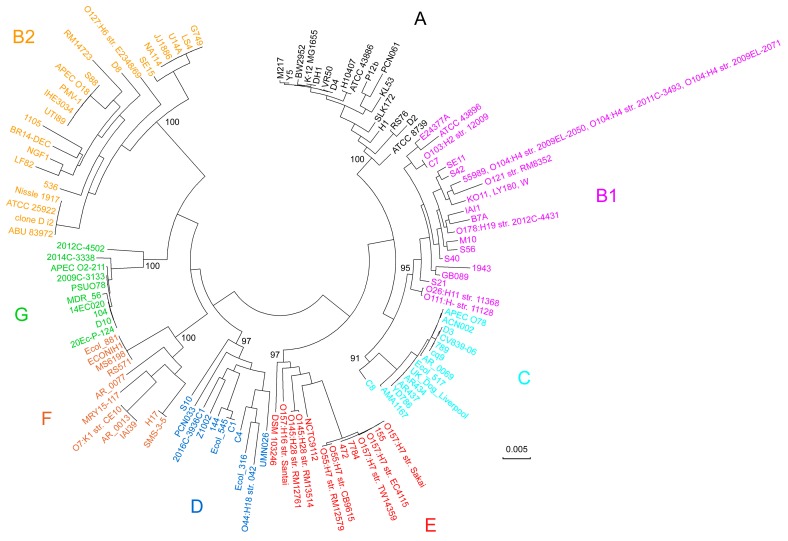
Phylogenetic tree for 124 *E. coli* strains inferred from concatenated aligned sequences of 27 genes in the IQ-TREE program [70] using the maximum likelihood method. The optimal model for nucleotide substitution was GTR+G+I (the general time-reversible model assuming a fixed portion of invariant sites and evolutionary rate differences described by the gamma-distribution). The branch support level shown in percentage was estimated based on 2000 iterations with ultrafast bootstrap approximation [71]. The scale bar corresponds to the number of nucleotide substitutions per site. The color code corresponds to eight indicated phylogroups. The names of all strains are indicated near corresponding branches and separated with comma for identical sequences in group B1.

The sequences of all 27 genes were aligned and concatenated as described in Materials and Methods. The phylogenetic tree was constructed with the IQ-TREE program [70] using the maximum likelihood method (Figure 2). All strains with previously known classification were correctly identified using this analysis, and all added genomes were distributed among the already known well-separated clades. Thus, we did not find signs of the existence of any additional phylogroup, but the sizes of the known groups increased significantly.

Probably the most important is the detection of 10 strains with complete genomes that form a clade between groups F and B2. Recently, this clade was classified into the separate phylogroup G based on shotgun sequences [46]. Another example is the phylogroup C. Previously, it was represented by only three strains with complete genomes (ACN002, APEC O78 and str. 789) [52,57,60]. It is also worth noting the apparent divergence of the groups D and F. Although the transfer of some strains from group D into a separate phylogroup F was proposed in 2008 [51], the feasibility of this was finally accepted only in 2013 [45], when six strains with either complete genomes or contigs were ascribed to it. The topology of the obtained tree was subsequently used as a reference for assessing the ability of the unique *k*-mers identified in the same 124 genomes to classify them into phylogroups. 

### 2.3. Phylotyping Based on Unique 18-mers and 22-mers Result in Identical Trees with the Same Topology as the Alignment-Based Approach

The availability of very large sets of marker sequences allows the efficient use of distance-based phylogenetic methods. Therefore, 124 sets containing unique 18-mers (or 22-mers) were obtained by UniSeq to evaluate the ability of the *k*-mer-based approach to distinguish eight phylogroups of *E. coli* and to assess the dependence of the distance-based method on *k*. Sets of unique 18- and 22-mers of *Escherichia albertii* KF1 were collected in the same way and used as outgroup samples. 

**Figure 3:**
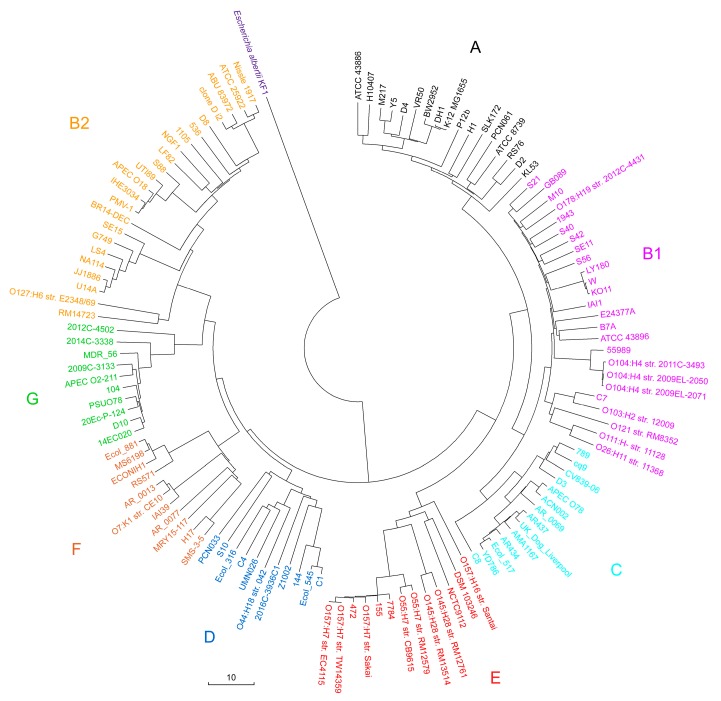
Phylogenetic tree constructed by the neighbor-joining method in the MEGA X program [73]. The tree was inferred from the pairwise distance matrix for 124 sets of 18-mers unique to the genera *Escherichia/Shigella* and was identical to the tree constructed on the basis of 22-mers. The set of marker 18-mers from the genome of *Escherichia albertii* KF1 was used as the outgroup sample. The scale bar shows the Sorensen distance as a percentage. The same color code as in Figure 2 denotes the clades of eight phylogroups.

Sorensen similarity indices (S) [72] for all marker sets were evaluated, and the corresponding distances (D) were calculated for all pairs of genomes using the formula: D = 1 – S. The created pairwise distance matrices were used to infer two phylogenetic trees with the neighbor-joining method, which turned out to be identical to each other, while the general topology of the new tree (Figure 3) appeared to be surprisingly similar to the topology of the previous one (Figure 2). Moreover, two branches in group B1, which had four (str. 55989, O104:H4 str. 2009EL-2050, O104:H4 str. 2009EL-2071 and O104:H4 str. 2011C-3493) and three (KO11, LY180, W) identical strains on the alignment-based tree (Figure 2), were divided in four and three individual leaves in the trees of Figure 3, respectively.

Although there are some differences in clades, for instance, strain PCN061 from group A was closer to P12b in the first case, but to ATCC 8,739 in the second, it became clear that *k*-mer-based phylogeny with different *k* can be used for accurate systematics of microorganisms, even at the intraspecies level. 

### 2.4. Phylogroup-Dependent Profiling of E. coli Presence in Human Intestinal Microbiomes

Although *Escherichia* strains can make a significant contribution to the production of certain proteins in the intestinal microbiome, they belong to the genus with a low abundance [74]. Therefore, the question of whether it is possible to track the presence of individual phylogroups in natural metagenomes was not trivial and was considered in this study. There were two ways to make such a taxonomic analysis. The easiest one was to use a “core” set of unique *k*-mers that are present in all genomes of the tested group and are absent in all other genomes in the database, including genomes of other *E. coli* phylogroups. However, any combination of genomes reduces the common set, and in some cases, this decrease is quite large. For example, the common set of unique 18-mers for the pair *E. coli* K-12 MG1655 and ETEC H10407 was 12.7% and 25.8% less than their individual sets (Figure 1b). When unique 22-mers were collected in 124 genomes using the reference database without all *E. coli* strains, and for each genome in the group only those sequences that were absent in other phylogroups were taken, we obtained individual sets of strain-specific markers ranging in size from 24,726 to 515,073 sequences (third and fourth columns in Table 1). In the core set of phylogroup B1 containing 25 genomes, only 143 sequences remained, although a combination of 10 genomes from group G yielded 51,125 sequences (Table 1). 

The inverse relationship between the number of strains in groups and the size of the core set of marker sequences is itself trivial, but the correlation between these values was not strong, and the B2 group with 23 genomes had the core set 126 times larger than phylogroup A with 17 genomes. This indicates a high dependence of the core set on the degree of evolutionary proximity of the strains in the group: the closest relatives have more common 22-mers (97.7–99.1% for the laboratory strains K-12 MG1655 and BW2952), than more distant ones, for example K-12 MG1655 and ETEC H10407 (Figure 1b). Thus, it became clear that a statistical analysis of the data obtained with such different-sized sets would not be convincing. 

The second way for taxonomical analysis was the use of cumulative sets, the size of which positively correlates (*R* = 0.55) with the number of genomes in groups (Table 1). In this case, all the unique 22-mers found in at least one genome in the group were combined, resulting in eight sets that were more comparable in size. It should be noted that the largest and smallest combined sets belong to groups B2 and C, respectively, which are not the largest and smallest groups in terms of their genome numbers. Reflecting different levels of genomic diversity in phylogroups, this also indicates a high heterogeneity of group B2, which includes mainly potentially pathogenic strains.

These sets were used to search for marker 22-mers in stool metagenomes from four healthy individuals and four patients with Crohn’s disease, which causes intestinal inflammation [75]. We took this collection of samples because in an original paper based on 16S rRNA typing of metagenomes from 27 healthy people and 121 patients with Crohn’s disease, it was found that only Enterobacteriaceae showed a significant increase in abundance specific to Crohn’s disease [75]. In that study, eleven shotgun metagenomes were also obtained to confirm the results of metabolomic analysis, and we took eight of them with the number of sequence reads ≥ 689004 to evaluate the degree of equilibrium between different phylogroup of *E. coli* in natural microflora.

The percentage of reads related to the species *E. coli* varied in eight metadata from 0.015% (metagenome SRX187525) to 2.74% (SRX187527). On average, this is 0.048% for healthy people, which corresponds to the knowledge of a low abundance of Enterobacteria in the intestinal microflora [76]. Table 2 shows the number of marker 22-mers found in different metagenomes for eight phylogroups. They vary from 462,763 (group B2 with the largest cumulative set in the average in size metagenome N8) to 0 (group G with a small cumulative set and in the smallest metagenome N4), which made it unreasonable to use smaller sets of shorter *k*-mers. In all samples obtained from patients with Crohn’s disease, *k*-mers from the group B2 were overrepresented. However, many detected 22-mers with a multiple presence in the metadata overlapped because they were not “primary” unique 22-mers, which complicates interpretation of the data obtained. Therefore, we collected all the sequence reads containing found 22-mers and used them for comparative analysis. Since the sequence reads are rarely identical, this reduced the risk of overestimating the presence of strains due to overlapping marker sequences. 

**Figure 4:**
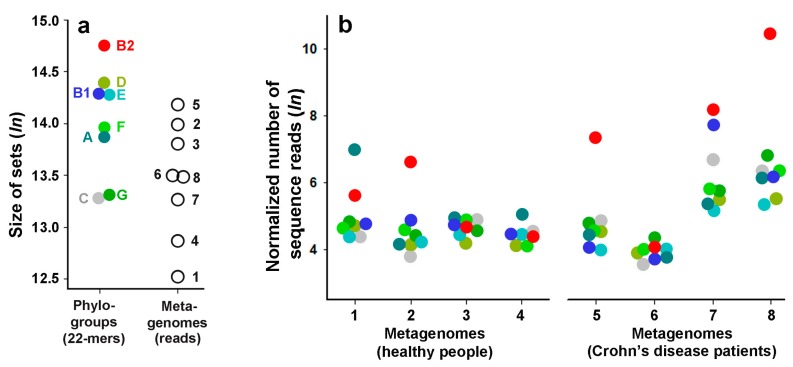
Phylogroup-dependent taxonomy of metagenomes from four healthy individuals (numbers 1–4) and four patients with Crohn’s disease (numbers 5–8). Panel (**a**) shows the size distribution for cumulative sets of unique 22-mers (colored symbols) and selected metagenomes numbered in the same way as in panel “**b**” (open symbols). Panel (**b**) demonstrates the number of sequence reads assigned to a particular group, normalized by the size of cumulative sets of 22-mers (Table 1) and the number of reads in metagenomes. Numerical values in both cases are presented as their natural logarithms.

After normalization to the size of the largest cumulative set (B2) of group-specific markers and to the size of the largest metagenome (N5) (Figure 4a), the natural logarithms of the number of sequence reads characterizing the presence of different groups in the eight selected metagenomes are plotted in Figure 4b. 

As a result, we detected representatives of all phylogroups in all metagenomes, except phylogroup G, which was absent in the smallest sample (SRX187518, N1). Bacteria from groups D, E, and F were found in approximately the same amount in all metagenomes. Groups B1 and C also fall in this category in healthy metagenomes, showing a higher presence in one disease-related sample (SRX187526, N7). Probably the most important observation made by this taxonomic analysis is the detection of the dominance for one or two phylogroups in metadata N2 (SRX187521), N3 (SRX187522), N5 (SRX187524), N7 (SRX187526), and N8 (SRX187527), clearly visible even on a logarithmic scale (Figure 4b). All three metagenomic samples that did not have a visible imbalance (SRX187518 (N1), SRX187523 (N4) and SRX187525 (N6)) have a relatively small number of marker 22-mers (Table 2). The low presence of *Escherichia* in these samples may indicate the inability of this taxon to compete with other bacteria, which impedes their growth. In the remaining five microbiomes, representatives of phylogroup B2 were overrepresented and even dominated in four samples, three of which were obtained from the patients with Crohn’s disease. However, the excessive presence of group B2 in sample N6, detected by the number of 22-mers (Table 2), disappeared when the number of sequence reads was used for comparison (Figure 4b), which currently does not allow us to consider this group as a disease-associated marker. 

## 3. Discussion

All the studies were based on the compact UniSeq software [44], which effectively detects unique *k*-mers in the tested genomes that are absent in the reference genomic database. In our case, the entire database contained 28540 nucleotide sequences of fully assembled bacterial genomes and plasmids, but the size of the reference database depended on the number of sequences belonging to the taxon of the tested genomes. Without *E. coli*, the list of sequences subjected to scanning included 26154 genomes or plasmids. When genera *Escherichia* and *Shigella* were ignored, 25853 sequences were scanned. The search time depended only on the number of scanned sequences and the speed of the hard disk and did not depend on the length of the tested genome or the length of *k*-mers, but the maximum value of *k* was limited by RAM. The program was tested in 32-bit OS and 4 Gb of RAM, which admits an increase in *k* up to 22. A feature of UniSeq is the use of original *k*-mer identifiers (Id), the computational time of which for a given genome does not depend on the value of *k*. When UniSeq searches for unique *k*-mers in the tested genome, it computes the Id of all *k*-mers in the list of target sequences without the need for their preliminary calculations and storage. 

Using UniSeq in this (Figure 1) and previous study [44], we evaluated the dependence of the number of unique *k*-mers on the value of *k* for the genomes of different taxa. Previously we observed that 17- and 18-mers gave maximum contribution to their number for the genomes of *E. cloacae* strains (SDM, EcWSU1, ENHKU01), *E. lignolyticus* SCF1, and *Cl. sporogenes* DSM 795, while the differential plot (ΔN/Δ*k*) for marker *k*-mers of *C. flavigena* DSM 20109 had a broad peak for 18 ≤ *k* ≤ 21 (Figure 1A in [44]). In this study, performed for various *E. coli* strains with an updated program and a significantly larger genomic database, the maximum increase in the number of strain-specific *k*-mers was detected in approximately the same range (18–20 n, Figure 1a). In part, this corresponds to the lengths (20, 25, and 31 n) used for comparative analysis in [42], although the 15-mers [41], and 30-31-mers [36,37,38,40] were also applied for taxonomic analysis. The choice of the *k* value is methodologically important and depends on the problem being solved. A relatively small number of short marker *k*-mers in the genomes may not be sufficient for taxonomy, while the limiting factor for phylogeny might be their diversity. However, our data show that sets of 18-mers, mainly consisting of “primary” unique sequences, have the same discriminative ability as sets of 22-mers, most of which are derived from shorter unique sequences. Biologically, this means that the plasticity of genomic sequences is already apparent at short distances.

We started this study in order to answer two questions: how sensitive is alignment-free phylogeny for intraspecies subtyping and how informative is intraspecies taxonomy based on marker *k*-mers? The expediency of the first question is justified by the global significance of *Escherichia coli* as a classic model organism for biology and evolution and as a potential pathogen in the microbiota of the human intestine. The extra-enteric pandemic strain(s) belonging to the serotype O104:H4 has already demonstrated the ability for aggressive expansion in human microbiomes [77,78]. Since the ecological niche of the strain and its pathogenicity largely depend on its evolutionary lineage, it would be useful to be able not only to identify already known pathogenic strains, but also to assess the risk of the appearance of active pathogens in human microbiota by the presence of pathogenicity-associated phylogroups. 

Intraspecies phylogroups of *E. coli* are ideal biological objects for assessing sensitivity of *k*-mer based phylogeny. The current classification includes eight phylogroups (see Introduction). However, it was accepted quite recently, and the rapid historical dynamic of the changes made assumed that new phylogroups of *E. coli* will still be discovered based on a larger set of complete genomes. However, in this study, we confirmed the current classification with eight phylogroups based on 124 genomes. Perhaps this is the most important achievement of the work, especially since the phylogenetic analysis was performed independently using the classical MLSA method (Figure 2) and sets of 18-mers unique to the genera *Escherichia/Shigella* (Figure 3). Phylogroups were determined for 65 additional strains, and it was found that alignment-free phylogeny allows quick and accurate classification of new isolates. 

The feasibility of the second question of how informative is the taxonomy based on strain-specific *k*-mers was justified by the fact that natural microbiomes contain thousands of different microorganisms, only a minor part of which are presented in the genomic sequences of NCBI database. Using classical phylotyping based on 16S RNA sequences, it is now easy to characterize the generic composition of microflora, but even species are identified with rather low accuracy [5]. However, pathogenic strains usually do not differ from non-pathogenic ones in 16S RNA sequences, and additional strain-specific markers are required to distinguish them. Since pathogenicity can be achieved by horizontal gene transfer or certain rearrangements in the genome that affect metabolic pathways but do not alter sequences, the ability to detect an unknown pathogenic strain based on “pathogenicity signatures” seems incredible. On the other hand, the advantage of any change in the genome of a particular bacterial cell is “checked” by the entire bacterial population, which either accepts or eliminates the mutant, based on the level of its suitability. Given that chronic Crohn’s disease is accompanied by persistent inflammation of the intestine, which is caused by specific bacterial communities, formed throughout the life of patients, it is likely that these communities are adapted to certain species, with or without a signature of pathogenicity, which support inflammatory homeostasis. Therefore, the question was: is there a bias towards phylogroups of *E. coli* containing many pathogenic strains in the microbiomes of sick people?

As a result, we found that all three metagenomes (N5, N7, and N8 in Figure 4b) with the largest presence of *E. coli* (0.13–2.74%) belong to sick people. This excess is mainly due to the presence of bacteria from the phylogroup B2, which includes 23 bacterial strains, 21 of which are conditional pathogens (Supplementary Table S1). This is what we expected to find; however, representatives of group B2 were also overrepresented in one healthy metagenome (N3 in Figure 4b), while one metagenome from a patient with Crohn’s disease show the dominance in only the number of 22-mers (Table 2). In the microbiome of a healthy person, the expansion of B2 bacteria can be caused by any other disease, while the absence of this expansion in a patient with Crohn’s disease can be explained by the inflammatory effect mediated by bacteria of some other genus. This cannot be discussed in the absence of clinical data that were not presented in the original paper [75]. Thus, we could not get a definite answer to the question posed. Equally unauthorized is the allegation of the dominance of representatives of group B2 in samples obtained from patients with Crohn’s disease, and the assertion that there is no disease-related difference. However, it should be noted that variations in the profile of dominant phylogroups in the metagenomes of different individuals have already been described and their analysis did not reveal significant correlations [79]. 

In any case, it became clear that 22-meric barcodes are sensitive taxonomic tools that can detect all phylogroups of *E. coli* even in relatively small libraries of sequence reads, despite the low prevalence of this taxon in human microflora. It also became clear that the profile of *E. coli* strains at the phylogroup level can be balanced, as in the cases of N4 and N6 metagenomes, or significantly unbalanced towards one or two groups (N2, N3, N5, N7 and N8) (Figure 4b). This is perhaps the most valuable observation in our taxonomic analysis, but the question of whether this is related to pathology should be addressed on the basis of a wider set of experimental data. 

## 4. Materials and Methods 

### 4.1. Database

A local copy of the NCBI GenBank database as of March 19, 2019 contained 28540 nucleotide sequences of fully assembled bacterial genomes and plasmids. This database included only those sequences in which the number of degenerated nucleotides did not exceed 5% of the length. All sequences were stored in digital form with the replacement of A, C, G, and T by 0, 1, 2, and 3, respectively, while each degenerated nucleotide S, W, R, Y, K, M, B, D, H, V, and N was changed to 4. To search for unique *k*-mers with *k* = 16, 18, 20, or 22 in 124 selected *E. coli* genomes in the case of comparative analysis (Figure 1) and *k*-mers for phylogenetic analysis (Figure 3), we created the list of target genomes, which included all sequences from the local database, besides bacterial chromosome and plasmid of the genera *Escherichia* and *Shigella*. Collecting the set of unique 22-mers for taxonomic analysis (Figure 4), only chromosomes and plasmids belonging to bacteria of the species *E. coli* were removed from the list of genomes in the local database. This minimized the risk of possible contribution given by other *Escherichia* or *Shigella* to the sets of detected phylogroup-specific 22-mers and sequence reads.

### 4.2. Outlines of UniSeq and Identification of “used” K-Mers 

We used the renewed version of UniSeq [44], which works in a 32-bit operating system, requires 4 Gb of RAM, and admits even *k*-mers with value of *k* up to 22. UniSeq “cyclizes” bacterial chromosomes and plasmids, regardless of whether they are really circular or not. For this, the 5′-terminal fragments of nucleotide sequences of the appropriate length were added to their 3′-ends. As in [35,37], when scanning UniSeq ignores *k*-mers containing ambiguous IUPAC symbol(s), encoded by the number 4.

The most important feature of UniSeq is the original way of identifying unique *k*-mers, which provides both compactness and high speed of the program. At the preparing stage, the FoundUse submodule scans input genome and computes the identifiers (Id) for all *k*-mers of specified length. For a given genome of length *L*, this requires a two-component array for hash codes and digital codes of *k*-mers (int h[L], int Cd[L], respectively), and array with indices (int Index [0xFFFFFFF + 1], where elements h[.], Cd[.]) and Index[.] occupy 2, 4, and 1 bytes, respectively. Integer *nu* is used to count *k*-mers. The detailed description of Id is given in Section 4.3. The logic of FoundUse is as follows:

1) while reading genome in the 3′→5′ direction, compute arrays (h[z’], Cd[z’]) for *k*-mers z’, which are complementary to the top strand;

2) *nu*=0; clear array Index;

3) while reading genome in the 5′→3′ direction, consider *k*-mer z in a given position p

a) compute(h[z], Cd(z)) for z;
b) if z is degenerated, then continue 3);
c) compute Id[p] that is min{(h[z’],Cd[z’]), (h[z],Cd(z)};
if(Index[Id[p].h] is not equal to 0) then go to 3; //because such Id was already registered//
else//save Id components of used *k*-mer at position p
{Index[Id[p].h]:=1; h[nu]:=Id[p].h]; Cd[nu]:=Id[p].Cd;
nu+=1;
}

4) write *nu* found *k*-mers that was saved in array (h[L],Cd[L]) to file RUsed;

According to the FoundUse logic, all *k*-mers in RUsed have different hashes h[.]. Therefore, among two *k-*mers with the same hash but different Cd, only the one with the leftmost position in the genome is registered. To reduce this loss, FoundUse also scans the complementary DNA strand and saves in IUsed those *k*-mers that do not repeat *k*-mers in RUsed. Despite this approach, some losses still remain due to repeated sequences in genome, but they do not exceed 3%.

### 4.3. Detection of Unique K-Mers in the Genomes

To detect unique *k*-mers among the used *k*-mers in the tested genome g, UniSeq scans the target set of genomes in the database, using RUsed and IUsed as two input files. By scanning both strands of genomes, the program computes the values Id[z]=(h[z],Cd(z)) for each *k*-mer z of target sequences. Once Id[z] is evaluated, the hash h[z] allows UniSeq to decide whether Id[z] is in RUsed or IUsed. If so, UniSeq marks Id[z] in RUsed/ IUsed as “not unique”.

Id identifiers were first computed for all non-degenerated 22-mers. Since our local database stores sequences in the digital form, each non-degenerated *q* is a numerical 22-component vector, where the components take values from 0 to 3. In the 5′→3′ direction, *q* is written as a numerical sequence, ***q*** = x_1_x_2_x_3_x_4_[z_1_z_2_...z_14_]y_1_y_2_y_3_y_4_, where the 14-mer in the brackets is the hash of q with the value h(q) = z_1_ 4^13^+z_2_ 4^12^+...+ z_14_ that occupies 28 bits of a 4-byte register. Hashes were computed by Horner’s rule [80] and gave the first component of Id(*q*).

The second component Cd(*q*) of Id(*q*) = (h(*q*),Cd(*q*)) was defined as Cd(q) = x_1_⋅4^7^ + y_1_⋅4^6^ + x_2_⋅4^5^ + y_2_⋅4^4^ + x_3_⋅4^3^ + y_3_⋅4^2^ + x_4_⋅4^1^ + y_4_. It occupies 2-byte register. Fast computing of Id identifiers was achieved by computing the Id(*x*) for current 22-mer *x* via Id(*q*) of the previous neighboring 22-mer *q* by using operator shift (“<<” in C++). To keep h(*x*) within 28 bit, the mask 0xFFFFFFF was used. For the identifier Id(*Q*), where *Q* is the complement of *q*, we calculated h(*Q*) and Cd(*Q)* following the same formulas but in the opposite direction to *q*.

Original hashing of 22-mers by means of their central 14-mers facilitates the identification of even *k*-mers with *k* < 22. Since all of them are parts of 22-mers and for each 16-20-mer there is a 22-mer that has it in the center, it is enough to use submodule FoundUse only to compute the identifiers of 22-mers. So, for example, for the 18-mer u=x_3_x_4_[z_1_z_2_...z_14_]y_1_y_2_, which is in the middle of the 22-mer q, Cd(q) is located in the register as follows: x_1_y_1_x_2_y_2_x_3_y_3_x_4_y_4_. Combining Cd(q) with mask=0011001111001100 gives 00y_1_00y_2_x_3_00x_4_00 and defines Id(u)=(h(q),Cd(q)&mask). As the mask is symmetric if inverted, it also works correctly for complement of u, providing Id(u’)=(h(q’),Cd(q’)&mask), where q’ is the complement of q. The numerical two component vectors Id(u) and Id(u’) are naturally ordered, permitting to define the identifier of *k*-mer u as Id(u)=min{id(u), id(u’)}. The *k*-mers of the test genome that were absent in the target sequences of the database were considered as “uniques”. Their identification in one genome takes a little less than 1 h if the target pool has the size of the current database and for interested researchers, it may be done on request.

### 4.4. Phylogenetic Inference

Several schemes have been used previously for multilocus phylotyping of *E. coli*. Fifteen conserved genes (*arcA*, *aroE*, *aspC*, *clpX*, *cyaA*, *dnaG*, *fadD*, *grpE*, *icd*, *lysP*, *mdh*, *mtlD*, *mutS*, *rpoS*, *uidA*) were applied by Qi et al. [81]. Eight genes (*dinB*, *icd*, *pabB*, *polB*, *putP*, *trpA*, *trpB*, *uidA*) were successively used by Jaureguy et al. [51]. Seven coding sequences (*adk*, *fumC*, *gyrB*, *icd*, *mdh*, *purA*, *recA*) were proposed for the phylogeny by Wirth et al. [82], and one (*aes*) was used in a paper by Lescat et al. [83]. However, only one gene (*icd*) was selected as a phylogenetic marker by three groups and two genes (*mdh* and *uidA*) were used in two studies. Combining all the above-mentioned phylotyping schemes, we got a set with 27 genes. Their nucleotide sequences were extracted from *E. coli* genomes in accordance with the NCBI GenBank annotation. Of the five orthologs that were either absent in annotations, or were indicated as two split genes, sequences for four were identified using NCBI BLAST (96.0–99.0% identity to the corresponding gene of *E. coli* MG1655 with 100% coverage). The fifth gene was not found in one genome and was indicated in the concatenated sequence by gaps. One truncated gene was evaluated in the same way. The sequence of one pseudogene was restored by elimination of insertion element (100% coverage, 99% identity). The names of all genes in all 124 genomes or coordinates of the found orthologs are given in Supplementary Table S1.

Twenty-seven independent alignments of 123–124 nucleotide sequences were obtained using the MUSCLE algorithm [84] and concatenated. For the resulting alignment, an optimal substitution model was found in the MEGA X [73] program with a built-in test using the Bayesian information criterion [85]. This model turned out to be GTR + G + I [86,87]. The phylogenetic tree was constructed in the IQ-TREE program [70] using the maximum likelihood method [88]. The level of branch support was estimated based on 2000 iterations of ultrafast bootstrap [71].

An alternative tree was obtained as follows. For each genome of 124 *E. coli* strains (Supplementary Table S1) and for the *E. albertii* KF1 genome (GenBank accession number CP007025.1), which was used as outgroup sample, sets of unique 18-mers were found that were present only in genera *Escherichia/Shigella*. Then a pairwise comparison of all sets was made and the Sorensen similarity indices were calculated [72]. A pairwise distance matrix was obtained and a phylogenetic tree was constructed using the neighbor-joining method [89] in MEGA X. Both trees were visualized in MEGA X.

### 4.5. E. coli Phylogroup Taxonomy of Metagenomic Data

For taxonomic analysis, eight metagenomes of the intestinal microbiota were selected. Obtained by shotgun sequencing on the Illumina MiSeq platform by Morgan et al. [70], these data included metagenomes of four healthy individuals (access numbers in the NCBI SRA: SRX187518, SRX187521, SRX187522, SRX187523) and four patients with Crohn’s disease (SRX187524, SRX187525, SRX187526, SRX187527). Fastq files downloaded from the NCBI SRA were filtered in Galaxy [90,91] using the “Filter by Quality” option (parameters: Q20 and coverage 90%), which removed reads containing less than 90% of bases sequenced with 99% accuracy.

Unique 22-mers were identified for each of 124 genomes and saved in separate files. Then, for each phylogroup A, B1, B2, C, E, D, F and G, identifiers of 22-mers belonging to all genomes of the given group were combined with deletion of copies. The search for the corresponding 22-mers in each metagenome was carried out using our UniTestExpress program. At the preparing stage, the auxiliary programs eliminated reads containing degenerated nucleotides and transcoded the entire metagenome into a numerical string in the same way as described above for genomes ({A, C, G, T}→{0,1,2,3}). Digital reads were then concatenated into a long numerical string, in which line break symbols were replaced with numerical 4. Such a prepared metagenomic string, together with each of the eight sets of phylogroup-specific identifiers, were the input files for UniTestExpress. This program operated with the metagenome in the same way that UniSeq worked with the genome, except that UniTestExpress looked for common 22-mers in input files, while UniSeq collected those *k*-mers of the test genome that were absent in the target genomes of the database.

The output UniTestExpress file displayed the set of marker *k*-mers found in the metagenome as the list of lines, each of which presented three characteristics X, Y, and Z. Of these, X was the nucleotide sequence of the 22-mer, whose identifier was found in the metagenome; Y was the number of its occurrence in the metagenome (shown in Table 2), and Z was the number of reads containing this 22-mer (used to prepare Figure 4).

### 4.6. Ethics Statement

All human data used in this study are from the NCBI BioProject ID number 175224, which is a publicly available database.

## Figures and Tables

**Table 1:** Statistics for phylogroup-specific sets of 22-mers for 124 genomes of *E. coli*.

Phylogroup	Number of Strains	Range in Size Variation for Sets of Marker 22-mers in Individual Genomes		Size of Core Sets	Size of Cumulative Sets
		Maximal	Minimal		
A	17	143,024	24,726	232	1,055,426
B1	25	161,117	72,365	143	1,600,260
B2	23	515,073	379,072	29,343	2,539,510
C	14	148,829	56,030	8444	586,272
D	11	368,049	243,470	1298	1,778,210
E	13	463,307	292,542	10,213	1,582,445
F	11	355,845	248,277	20,640	1,159,521
G	10	235,711	146,632	51,125	599,863

**Table 2:** Number of *E. coli* group-specific 22-mers, found in selected metagenomes.

SRA ID of Metagenome (N)	A	B1	B2	C	D	E	F	G
**Samples of healthy individuals**								
SRX187518 (1)	17	15	26	5	12	14	8	0
SRX187521 (2)	9382	223	5060	33	559	110	182	39
SRX187522 (3)	31	191	11,566	17	34	273	83	15
SRX187523 (4)	29	29	62	10	21	22	17	6
**Samples of Crohn’s Disease Patients**								
SRX187524 (5)	105	307	36,698	64	212	54	279	305
SRX187525 (6)	10	28	81	6	22	24	23	15
SRX187526 (7)	211	11388	38,389	3435	774	223	1147	420
SRX187527 (8)	944	4418	462,763	1292	1019	1104	1713	2024

## Supplementary Materials

Supplementary materials can be found at https://www.mdpi.com/1422-0067/21/3/944/s1.
